# Ginsenoside Rg1 ameliorates the cognitive deficits in D-galactose and AlCl_3_-induced aging mice by restoring FGF2-Akt and BDNF-TrkB signaling axis to inhibit apoptosis

**DOI:** 10.7150/ijms.43979

**Published:** 2020-04-15

**Authors:** Si-Jia Zhong, Lin Wang, Run-Ze Gu, Wen-Hao Zhang, Rongfeng Lan, Xiao-Yan Qin

**Affiliations:** 1 Center on Translational Neuroscience, College of Life and Environmental Sciences, Minzu University of China, Beijing 100081, China.; 2 Department of Cell Biology & Medical Genetics, School of Basic Medical Sciences, Shenzhen University Health Science Center, Shenzhen 518060, China.; 3 College of Economics and management, North China Electric Power University, Beijing 102206, China.

**Keywords:** estradiol, ginsenoside Rg1, learning and memory, Morris water maze, step down avoidance test

## Abstract

Ginsenoside Rg1 is the main active ingredient of *Panax ginseng* with the activity of neuroprotective, antioxidant and strengthening the immune system. Therefore, we hypothesized that Rg1 may afford anti-aging effects although the mechanism remains to be elucidated. In this study, chemically induced aging mice were established by consecutive administration of D-galactose and AlCl_3_. We found that Rg1 effectively ameliorates spatial learning and memory deficits in aging mice demonstrated by their improved performance in step down avoidance tests and Morris water maze experiments. Rg1 restored aging-induced decline of FGF2 and BDNF, reactivated TrkB/Akt signaling pathways in the hippocampus and prefrontal cortex to inhibit apoptosis, for the expression of anti-apoptotic protein Bcl-2 and apoptosis promoting enzyme cleaved-Caspase3 were antagonistically restored. Therefore, these results established the anti-aging effects of Rg1, and FGF2, BDNF and associated signaling pathways might be promising targets. Our data may provide a new avenue to the pharmacological research and diet therapeutic role of ethnic products such as Rg1 in anti-aging and aging associated diseases.

## Introduction

*Panax ginseng* is a kind of perennial plant belonging to *Araliaceae*, which is known as the king of herbs in eastern Asia. It has a long history been used as tonic with the properties of tranquilizing mind, improving intelligence, relieving cough, nourishing and strengthening body and delaying aging. Ginseng has been used as a food and ethnic medicine in China for centuries, for it is edible and easy to add to the diet such as stewed in water to make tea or added to recipes such as stir-fry and soup. Ginsenoside Rg1 is the most abundant ginsenoside identified in *Panax ginseng* and demonstrates the neuroprotective, antidepressant and antioxidant properties and estrogen-like activity in cell culture and animal models 1-3. Rg1 have the capacity to regulate neurotransmitters, activate the learning and memory related signal pathways, and enhance synaptic plasticity and the long-term potentiation of postsynaptic potentials in the brain regions such as cortex and hippocampus 4, 5. Accordingly, the effects of Rg1 were emphasized on improving the learning and memory in rodent models of senescence, estrogen deficiency and cerebral ischemia and reperfusion 5. Therefore, it is well established that Rg1 affords neuroprotective effects and could be used in treating neurodegenerative diseases such as Alzheimer's and Parkinson' diseases. However, the molecular mechanism regarding of ginsenoside Rg1 to exert the above functions remains elucidated.

Fibroblast growth factor (FGFs) is a large family of polypeptide mitogens with similar structure and molecular weight (17-34 kDa). FGFs play an important role in the development of the central nervous system, involving the proliferation of neural stem cells, neurogenesis, axon growth and differentiation, etc. 6. FGF mediated signaling encourages the growth of the developing cortex, and allows the astrocyte self-renewal 7. Especially, FGF2 is emphasized for its mediated activation of PI3K/Akt signaling axis to inhibit apoptosis and strengthen neuron survival, neurogenesis and nerve repair 8-10. Akt is a serine/threonine kinase, which is attracted to the cell membrane through the interaction of phosphoinositol docking sites resulting in it fully activation. Activated Akt mediates downstream reactions by phosphorylating a series of intracellular proteins, including cell growth, survival, proliferation and differentiation 11. In the nucleus, Akt enhances the transcription of anti-apoptotic genes such as Bcl-2 but inhibits the transcription factors promoting the expression of cell apoptosis-related genes. Moderate activity and healthy response of FGF2-PI3K/Akt signaling axis are essential for neural homeostasis and functions 12.

From the aforementioned, we hypothesized that ginsenoside Rg1 may exert the anti-aging effects. To resolve this hypothesis, we introduced chemically induced aging mice and the effects of Rg1 on spatial learning and memory ability were assessed by behavioral procedures such as step down avoidance tests and Morris water maze experiments. The functions of FGF2-Akt and BNDF-TrkB signaling pathways in Rg1-mediated anti-aging effects have been emphasized, for these pathways contribute to various aspects of neural functions, such as maintaining the integrity of neurons or neurite outgrow, promoting synaptic plasticity and stability, anti-apoptosis, and anti-toxic inflammation. Therefore, the expression of FGF2, BDNF and the status of Akt/TrkB signaling axis was focused in Rg1 mediated anti-aging functions.

## Materials and Methods

### Animals

Male Kunming mice (3~4 weeks) were obtained from Beijing Vital River Laboratory Animal Technology Co., Ltd. and kept in a temperature and humidity-controlled room under a 12 h light/dark cycle with free access to diet and drinking water. The experimental procedures were performed in accordance with the Guidelines for Care and Use of Laboratory Animals of Beijing Municipality and approved by the Animal Care and Use Committee of the College of Life and Environmental Sciences, Minzu University of China.

Chemically induced aging mice were established as described previously 13. In brief, mice were randomly divided into five groups after adaptation according to Table 1. Mice were injected subcutaneously of D-Gal (150 mg·kg^-1^) and intragastrically administered with AlCl_3_ (13 mg·kg^-1^) once a day for 42 days, whereas their counterparts (the normal control) received equal amounts of 0.9% saline instead. After modeling, aging model mice were intraperitoneally injected with optimized concentrations of Rg1 10 mg·kg^-1^ (group Rg1-L, low dose), 20 mg·kg^-1^ (group Rg1-H, high dose) 14 as experimental treatments, estradiol (0.1 mg·kg^-1^) as a positive control and saline as a drug-free control. Estradiol (E2) is an estrogen steroid hormone that was established as an effective regulator of cognitive functions 15-17. Meanwhile, mice in the normal control group were administered 0.9% saline.

### Behavioral procedures

Step down avoidance (SDA) test. The inhibitory avoidance apparatus was a wooden box (five rooms, each room 12×12×18 cm), the bottom of which consists of parallel stainless steel rods, a wooden platform was placed in the center of the grid floor. In the training process, the animal is first placed on the platform. When stepping on the power grid, it was immediately shocked (0.6 mA, 30V). The electric shock is transmitted to the grid floor through an isolated stimulator (YLS-3 TB, Weixin Star Technology Co. Ltd. Beijing). The test was conducted 2 h after training. The procedure was the same as the training, except that there was no electric shock. Mouse was placed on the platform and recorded the numbers of avoidance errors within 300 s.

Morris water maze (MWM) experiment. The water maze consisting of a circular pool (120 cm diameter × 50 cm high) filled with opaque water to a depth of 30 cm (25 ± 1 °C) was settled as previously 18. An escape platform (10 cm×10 cm) was placed in one quadrant and submerged under water surface for 1 cm. In the positioning navigation training experiments, the animal was placed in different quadrants every day and was allowed to find the platform freely. If the platform is not found within 1 min, the animal would be guided to find the platform manually. The time required by the animal to find the platform is recorded as latency time. On the sixth day, space exploration test was carried out in which the escape platform was removed, the animal was placed in the opposite to the quadrant where the platform was located, and the time that the animal stayed in the quadrant of the platform had originally placed within 1 min was recorded, as well as the motion track and the aggregation degree of hot spots in space exploration. The longer the animal stay in the quadrant (residence time) where the original escape platform is located, the more obvious the purpose of the trajectory (distance moved) and the more hot spots are gathered are both indicate the stronger learning and memory ability of the animal.

### Chemicals

Ginsenoside Rg1 (#CAS22427-39-0, HPLC purity >98%) was provided by Wuhan Zhongchang Guoyan Standard Technology. Co., Ltd. with a license of No. MUST-17021446. D-Gal (#G0750), AlCl_3_ (#563919) and estradiol (E2) (#E2758) were purchased from Sigma-Aldrich, LLC. Rg1, D-Gal and AlCl_3_ were dissolved in 0.9% saline. Estradiol was dissolved in distilled water supplemented with 2% ethanol.

### Western blotting

Western blotting of hippocampal and prefrontal cortex proteins was performed as described previously 12, 19. The antibodies used in the experiments were included as following, Goat anti-mouse IgG-HRP (#ZDR-5307, dilution 1:5000) and goat anti-rabbit IgG-HRP (#ZDR-5306, dilution 1:5000) antibodies were purchased from ZSGB-BIO. Anti-Akt (#9272, dilution 1:1000), p-Akt (Ser473) (#4060, dilution 1:1000), Bcl-2 (#3498, dilution 1:5000), BDNF (#47808, dilution 1:1000), TrkB (#4606, dilution 1:1000), p-TrkB (#4619, dilution 1:1000), Cleaved- Caspase3 (#9661, dilution 1:1000) and β-actin (#4970, dilution 1:3000) antibodies were provided by Cell Signaling Technology, Inc. Anti fibroblast growth factor 2 (FGF2) (#sc-136255, dilution 1:1000) antibody was provided by Santa Cruz Biotechnology, Inc. The expression of the target protein was detected and photographed in the Odyssey CLx infrared fluorescence imaging system (LI-COR Biosciences). The relative protein expression levels are quantified by the density-value of the bands and normalized to loading control.

### Statistical analysis

Data were presented as mean±s.e.m. One-way ANOVA with Dunnett's multiple comparison tests were performed to compare the variance of significance. Graphs were prepared by GraphPad Prism 8.0 (GraphPad Software).

## Results

### Establishment of chemically induced aging mice models

**Figure 1:**
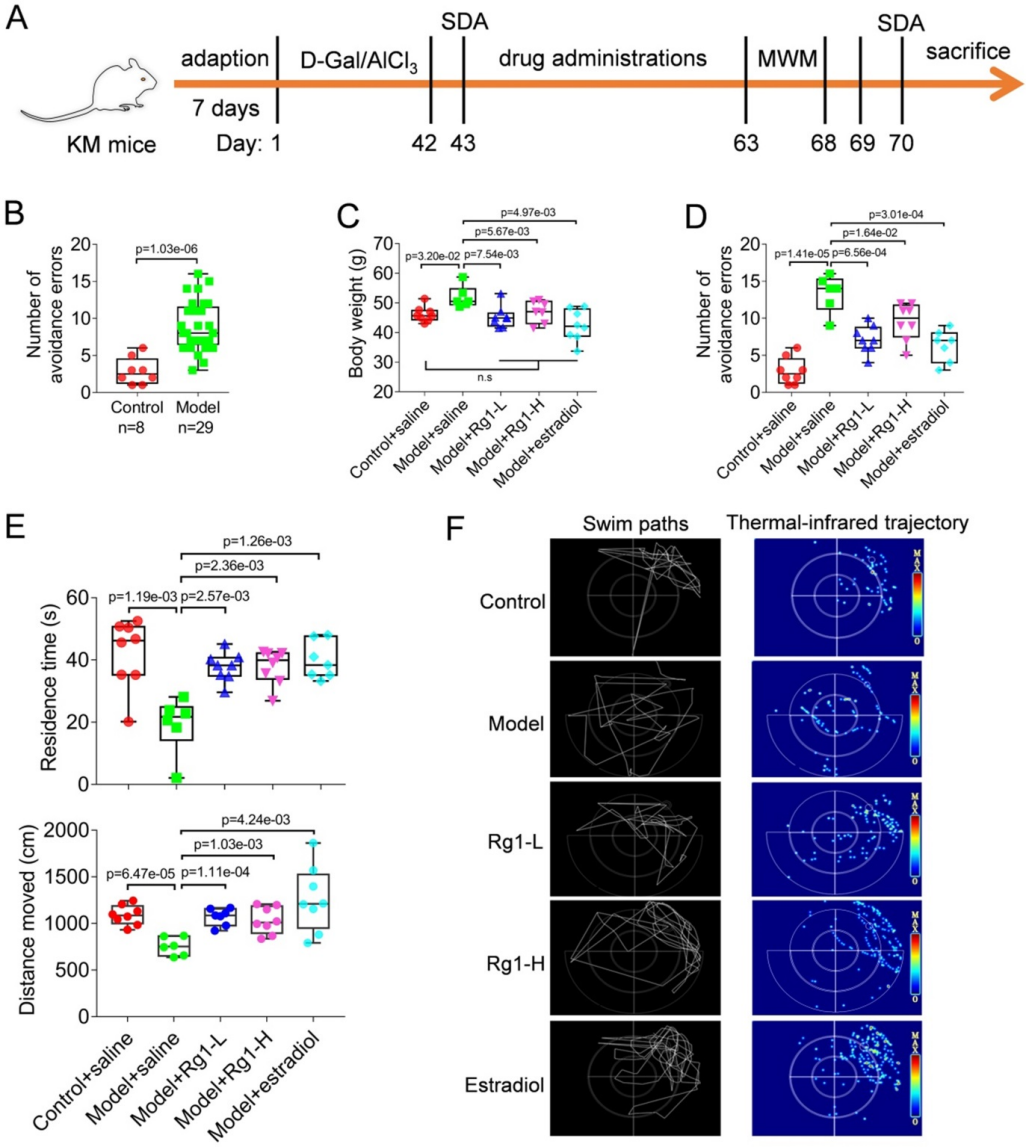
** Rg1 effectively ameliorates the cognitive deficits in chemically induced aging mice. (A)** Timeline of the experimental flow to establish chemically induced aging mice models through the administration of D-Gal (150 mg·kg^-1^) combined with AlCl_3_ (13 mg·kg^-1^) and Rg1 treatment. **(B)** Establishment of chemically induced aging mice that evaluated by step down avoidance (SDA) tests. Modeled aging mice showed a significant increase in the number of avoidance errors than normal control counterparts. **(C)** Chemically induced aging increased the body weight of animals, whereas Rg1 ameliorated the aging symptoms and restored the weight to normal. Data of repeated trials were showed by points in the box and whiskers plot. **(D)** Rg1 effectively improved the behavioral performance of aging mice in SDA test with reduced avoidance errors. Rg1-L, ginsenoside Rg1 low dose (10 mg· kg-1); Rg1-H, ginsenoside Rg1 high dose (20 mg· kg-1). **(E)** Rg1 effectively improved the performance of the aging mice in the Morris water maze (MWM) tests. Residence time and distance moved indicated the time or distance, respectively, the animal stayed in the target quadrant where the escape platform had been removed. **(F)** Representative swim paths and thermal-infrared trajectory in MWM tests. Aging mice treated with Rg1 or estradiol showed enriched locations in the quadrant of the escape platform had originally placed compared with the group treated with saline. In this figure, the statistical significance of intergroup differences is performed by one-way ANOVA and Dunnett's multiple comparisons test. Corrected p values were provided, n.s indicates non-significant.

To establish animal models, male Kunming mice were grouped (Table 1) and administered with D-Gal (150 mg·kg^-1^) plus AlCl_3_ (13 mg·kg^-1^) once per day, as indicated in the timeline (Figure 1A) and *Materials and methods* as described previously 13. After completion of modeling, the spatial learning and memory ability in mice were examined by behavioral procedure--step down avoidance test (SDA). Chemically induced aging mice made more errors in avoiding electric shock than that of healthy controls (Figure 1B), indicating significant cognitive deficits in the animals. Therefore, we successfully established the aging mouse models by consecutive 42 days-medication of D-Gal and AlCl_3_.

### Rg1 effectively ameliorates the cognitive deficits of chemically induced aging mice

The anti-aging effects of Rg1 were determined in D-Gal and AlCl_3_-induced model mice. Rg1 was intraperitoneally injected into mice with low and high doses as described in *Materials and methods.* Aging mice showed a significant increase in body weight due to aging induced reduction of exercise. However, Rg1 treatments afforded anti-aging effects and restored the weight to normal (Figure 1C). The SDA tests were repeated after the completion of Rg1 administrations. Consistently, we found that Rg1 significantly ameliorated the cognitive deficits of aging mice, for the numbers of avoidance errors were remarkably reduced (Figure 1D). Meanwhile, the conditions in the positive control group showed similar results, for estradiol effectively restored the cognitive impairments of aging mice (Figure 1C). In addition, both medications of Rg1 and estradiol improved the behavioral performance of aging mice in positioning navigation training (MWM tests). Animals in Rg1 and estradiol treated groups spent less latency time in comparison with their saline-treated counterparts (Table 2). Consistently, in the followed space exploration tests, Rg1 or estradiol significantly improved the performance of aging mice that the animals retained longer time and produced more gathered paths in the target quadrant, where the escape platform had originally plated (Figure 1 E). In contrast, aging mice treated with saline retained the cognitive deficits compared with the normal control groups. According to the swim paths and thermal- infrared trajectories, aging mice (model+saline group) showed obvious scattered track indicating impairment of cognitive ability (Figure 1F). The trajectories of Rg1 or estradiol-treated mice showed an obvious purpose and concentrated hot spots in the target quadrant (Figure 1F), indicating the recovery of their spatial learning and memory. These results suggested that Rg1 can effectively improve the cognitive deficits in chemically induced aging mice.

### Rg1 effectively restores the FGF2-Akt and BDNF-TrkB signaling pathways in the hippocampus and prefrontal cortex to inhibit neuronal apoptosis and ameliorate cognitive deficits

**Figure 2:**
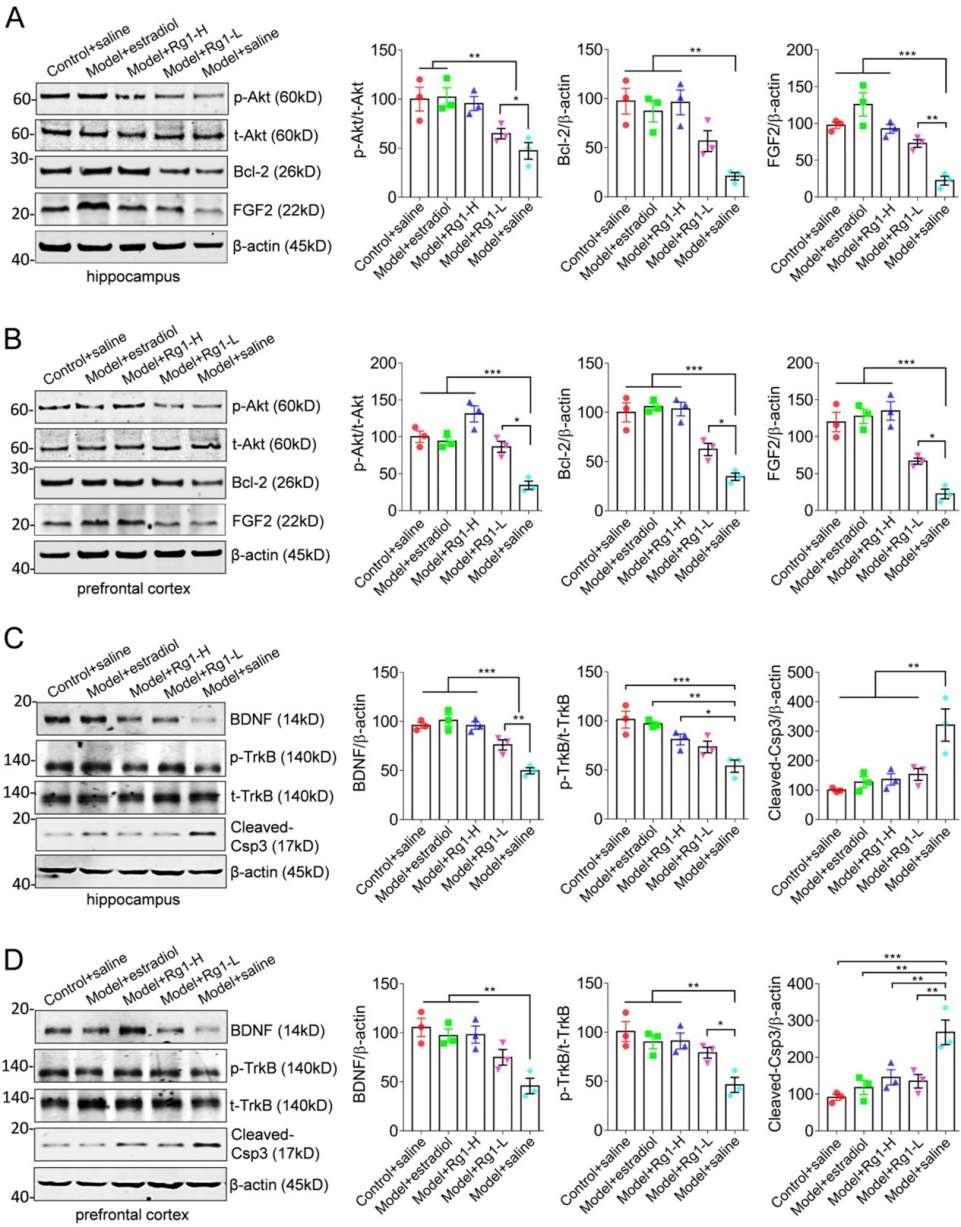
** Rg1 reactivates the expression of FGF2, BDNF and associated TrkB-Akt and Bcl-2/caspase3 pathways in the hippocampus and prefrontal cortex.** (A-B) Representative Western blotting showed the rescue of FGF2 expression and the recovery of Akt activation in the hippocampus and prefrontal cortex of aging mice by Rg1 medication. Consistently, the expression of anti-apoptotic protein Bcl-2 was reemerged to promote cell survival. (C-D) Representative blots showed the rescue of BDNF expression and TrkB activation through Rg1 administration. Proteins expression levels were semi-quantitative analyzed and normalized. Statistical variance of significance was performed by one-way ANOVA and Dunnett's multiple comparisons test. *p <0.05, **p < 0.01, ***p < 0.001.

To investigate whether the FGF2-Akt, BDNF- TrkB signaling pathways participate in the anti-aging functions of Rg1, we examined the expression of FGF2 and BDNF, the phosphorylation status of Akt or TrkB and apoptosis-associated proteins Bcl-2 and cleaved- Caspase3 in the hippocampus and prefrontal cortex (Figure 2). The expression of FGF2, BDNF, p-Akt, p-TrkB and Bcl-2 was remarkably reduced in chemically induced aging mice. In contrast, the expression of apoptosis promoting enzyme cleaved- caspasee3 was obviously elevated (Figure 2, the blots of model+saline). Thus, chemically induced aging was characterized by biochemistry impairment of chemokine or neurotrophins, and associated signaling pathways. However, the administration of Rg1 or estradiol rescued the expression of FGF2 and BDNF, and reactivated Akt and TrkB, suggesting the restoration of FGF2-Akt and BDNF-TrkB signaling pathways (Figure 2, the sets of Rg1-L, Rg1-H and estradiol). Similarly, the expression of apoptosis associated Bcl-2 and cleaved-Caspase3 were antagonistically restored (Figure 2). Therefore, we concluded that chemically induced aging impaired the expression of FGF2, BDNF and associated signaling pathways in the hippocampus and prefrontal cortex. The administration of Rg1 effectively restored the biochemistry of FGF2-Akt and BDNF-TrkB signaling pathways to exert anti-aging functions.

## Discussion

**Figure 3:**
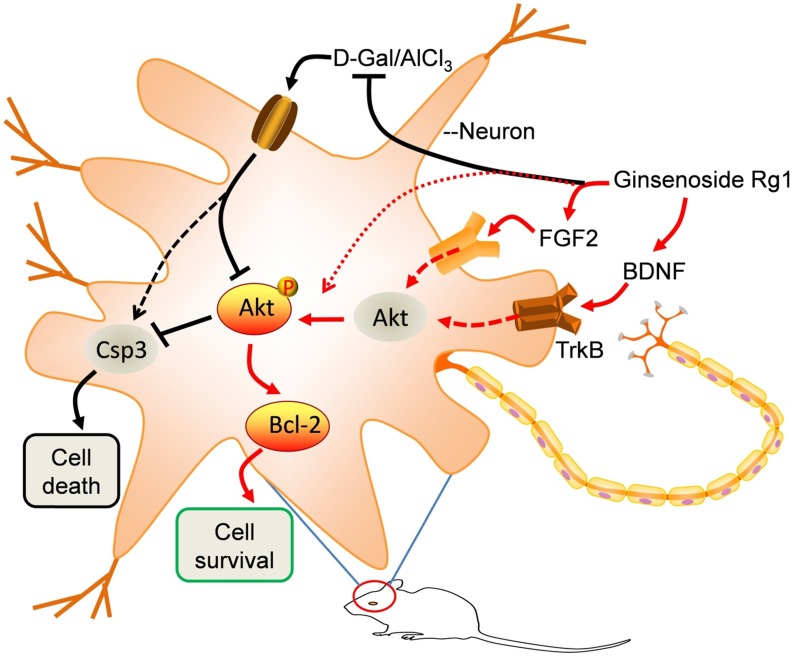
** A schematic model illustrates the anti-aging effects of Rg1.** In D-Gal/AlCl_3_ induced aging mice, the FGF2-Akt, BDNF-TrkB and Bcl-2 signaling pathways were obviously inhibited, resulting in apoptotic caspase3 activation, neural damage and impairment of spatial learning and memory functions. Rg1 rescued the decline of FGF2 and BDNF, reactivated their associated signaling pathways to afford neuroprotective functions.

Learning and memory deficits are common symptoms of the elderly, and it is an important sign of brain aging 20. Hippocampus is the hub of learning and memory in the brain. Aging related cell apoptosis and loss in the hippocampus will directly cause learning and memory deficits 21. By contrast, the prefrontal cortex is the most complex and least understood area where all kinds of high-level psychological activities, such as consciousness, thinking, and imagination are occurring 22. The functional impairment in this area is related to various neurological, mental and psychological diseases such as schizophrenia 23, Parkinson's disease 24 and attention-deficit hyperactivity disorder 25. In anti-aging pharmacological studies, D-Gal/AlCl_3_ induced aging models have been extensively used 26-28. D-Gal produces glycation end-product *in vivo* and induces toxicity to accelerate cell apoptosis, whereas AlCl_3_ is a neurotoxin to induce the overexpression of APP (amyloid precursor protein) in neurons and the production of toxic Aβ (amyloid beta). Alternatively, astrocyte senescence may also contribute to cognitive decline 29, 30. The energy powering and redox homeostasis maintenance supported by neurovascular coupling is also essential for healthy cognitive function, for the supplement of redox donor or restoration of mitochondrium's activities might improve cognitive function 31, 32. Therefore, consecutive administration of D-Gal and AlCl_3_ induced toxicity accelerates aging and seriously impair learning and memory 33. However, in D-Gal induced rodent aging models, ginsenoside Rg1 afforded anti-oxidant and anti-inflammatory effects to prevent cognitive impairment and hippocampal cell senescence, in which the Akt/mTOR signaling was inhibited 34-36. However, compelling evidence has supported the longevity effect through the inhibition of mTOR activity 37. Therefore, a moderate activity of Akt is required to prevent neural apoptosis and anti-toxic inflammation 12. In other words, neurotrophins such as BDNF (brain-derived neurotrophic factor) or chemokine like FGF2 activated TrkB and Akt signaling axis to inhibit apoptosis and preserve the integrity of neurons and synapse plasticity is essential for brain functions. In this work, we confirmed the anti-aging effects of Rg1 in D-Gal/AlCl_3_ induced aging mice that Rg1 restored the biochemistry of FGF2, BDNF and associated signaling axis (Figure 3). Moreover, Rg1 was reported to ameliorate the behavioral abnormalities and modulates the hippocampal proteomics in triple transgenic female Alzheimer's mice (3xTg-AD) 38. It is interesting to decipher the mechanistic avenue of how Rg1 to rescue the expression of FGF2 and BDNF in aging mice, especially in the hippocampus and prefrontal cortex, for chemokine or neurotrophins exerted extraordinary functions in the maintenance of neural activities 3. One mechanism of action has been suggested for Rg1, which is based on its similarity to steroid hormones and can be agonist of its receptors 39, 40. Estradiol is considered to afford neuroprotective functions and enhance learning and memory by activating intercellular signaling pathways^15^. Therefore, Rg1 can be a promising estrogen- like molecule to modulate neural homeostasis and brain functions, especially in aging and age-related diseases. For example, anti-Alzheimer's disease drugs in clinic trials such as donepezil, galantamine and memantine can only moderately alleviate the symptoms of patients but exert serious toxic and side-effects on the liver and kidney 41-43. In contrast, Rg1 is a nontoxic natural product and has already been established for renal, cardiovascular and hepatic-protective functions in various cells and animal models 14, 44, 45. Consistently, we observed that the learning and spatial memory deficits in aging mice could be restored by Rg1 administration in a dose-independent manner (Figure 1 and Figure 2). Either the administration of ginsenoside Rg1 or estradiol can recover the expression of FGF2, BDNF, and their associated signaling pathways in the hippocampus and prefrontal cortex (Figure 2). In summary, ginsenoside Rg1 is established for its activity in ameliorating cognitive deficits in chemically induced aging mice. FGF2-Akt and BDNF-TrkB signaling pathways were reactivated by Rg1 in the hippocampus and prefrontal cortex to inhibit neuronal apoptosis and prevent cognitive deficits. Collectively, this study will provide an experimental basis for the development and use of ginsenoside for anti-aging and age-related diseases.

## Figures and Tables

**Table 1:** Animal grouping and drug administrations.

Groups	D-Gal (150 mg·kg^-1^/day) AlCl_3_ (13 mg·kg^-1^/day)	Drug Treatment	Dose (mg·kg^-1^)
Control (n=8)	-	saline	10
Model (n=6)	+	saline	10
Rg1-L (n=7)	+	ginsenoside Rg1 low dose	10
Rg1-H (n=8)	+	ginsenoside Rg1 high dose	20
Estradiol (n=8)	+	estradiol	10

**Table 2:** The latency time of Morris water maze navigation experiment in mice.

Group	Latency time (s)				
	Day 1	Day 2	Day 3	Day 4	Day 5
Control (n=8)	42.08±12.12	37.94±10.96	36.83±7.51	29.11±9.65	31.63±2.86
Model (n=6)	50.56±8.94**	45.74±9.74**	38.04±4.78	34.53±9.22*	33.13±2.86*
Rg1-L (n=7)	44.05±11.51^#^	35.97±13.17^#^	29.14±5.62^##^	28.18±6.32^#^	25.99±6.77^#^
Rg1-H (n=8)	42.78±6.55^#^	32.17±9.00^##^	30.90±12.19^#^	30.05±3.21^#^	26.51±4.73^##^
Estradiol (n=8)	46.19±7.49^#^	36.93±12.18^#^	29.99±9.86^#^	28.97±7.77^#^	28.03±5.87^#^

Values are mean ± standard deviations. Rg1-L, ginsenoside Rg1 low dose; Rg1-H, ginsenoside Rg1 high dose. *Significance from control indicator of corresponding sets of experiments.*, p<0.05, **, p<0.01. #Significance from model indicator of corresponding sets of experiments. #, p<0.05, ##, p<0.01.

## Abbreviations

Aβ: amyloid beta
APP: amyloid precursor protein
BDNF: Brain-derived neurotrophic factor
FGF2: fibroblast growth factor 2
MWM: Morris water maze
SDA: step down avoidance

## References

[1] Choi JR, Hong SW, Kim Y, Jang SE, Kim NJ, Han MJ (2011). Metabolic activities of ginseng and its constituents, ginsenoside rb1 and rg1, by human intestinal microflora. J Ginseng Res.

[2] Dang H, Chen Y, Liu X, Wang Q, Wang L, Jia W (2009). Antidepressant effects of ginseng total saponins in the forced swimming test and chronic mild stress models of depression. Prog Neuropsychopharmacol Biol Psychiatry.

[3] Liu Z, Qi Y, Cheng Z, Zhu X, Fan C, Yu SY (2016). The effects of ginsenoside Rg1 on chronic stress induced depression-like behaviors, BDNF expression and the phosphorylation of PKA and CREB in rats. Neuroscience.

[4] Shen LH, Zhang JT (2004). Ginsenoside Rg1 promotes proliferation of hippocampal progenitor cells. Neurol Res.

[5] Gong L, Li SL, Li H, Zhang L (2011). Ginsenoside Rg1 protects primary cultured rat hippocampal neurons from cell apoptosis induced by beta-amyloid protein. Pharm Biol.

[6] Yoshimura S, Takagi Y, Harada J, Teramoto T, Thomas SS, Waeber C (2001). FGF-2 regulation of neurogenesis in adult hippocampus after brain injury. Proc Natl Acad Sci U S A.

[7] Nagayasu T, Miyata S, Hayashi N, Takano R, Kariya Y, Kamei K (2005). Heparin structures in FGF-2-dependent morphological transformation of astrocytes. J Biomed Mater Res A.

[8] Agas D, Marchetti L, Menghi G, Materazzi S, Materazzi G, Capacchietti M (2008). Anti-apoptotic Bcl-2 enhancing requires FGF-2/FGF receptor 1 binding in mouse osteoblasts. J Cell Physiol.

[9] Wang L, Li XX, Chen X, Qin XY, Kardami E, Cheng Y (2018). Antidepressant-Like Effects of Low- and High-Molecular Weight FGF-2 on Chronic Unpredictable Mild Stress Mice. Front Mol Neurosci.

[10] Allodi I, Mecollari V, Gonzalez-Perez F, Eggers R, Hoyng S, Verhaagen J (2014). Schwann cells transduced with a lentiviral vector encoding Fgf-2 promote motor neuron regeneration following sciatic nerve injury. Glia.

[11] Manning BD, Toker A (2017). AKT/PKB Signaling: Navigating the Network. Cell.

[12] Zhong SJ, Wang L, Wu HT, Lan R, Qin XY (2019). Coeloglossum viride var. bracteatum extract improves learning and memory of chemically-induced aging mice through upregulating neurotrophins BDNF and FGF2 and sequestering neuroinflammation. J Funct Foods.

[13] Xiao F, Li XG, Zhang XY, Hou JD, Lin LF, Gao Q (2011). Combined administration of D-galactose and aluminium induces Alzheimer-like lesions in brain. Neurosci Bull.

[14] Zhao Q, Yang M, Deng Y, Yu H, Wang L, Teng F (2015). The Safety Evaluation of Salvianolic Acid B and Ginsenoside Rg1 Combination on Mice. Int J Mol Sci.

[15] Luine VN (2014). Estradiol and cognitive function: past, present and future. Horm Behav.

[16] Aenlle KK, Foster TC (2010). Aging alters the expression of genes for neuroprotection and synaptic function following acute estradiol treatment. Hippocampus.

[17] Bohacek J, Daniel JM (2009). The ability of oestradiol administration to regulate protein levels of oestrogen receptor alpha in the hippocampus and prefrontal cortex of middle-aged rats is altered following long-term ovarian hormone deprivation. J Neuroendocrinol.

[18] Morris RG, Garrud P, Rawlins JN, O'Keefe J (1982). Place navigation impaired in rats with hippocampal lesions. Nature.

[19] Pan RY, Ma J, Wu HT, Liu QS, Qin XY, Cheng Y (2017). Neuroprotective effects of a Coeloglossum viride var. Bracteatum extract in vitro and in vivo. Sci Rep.

[20] Wadsworth LP, Lorius N, Donovan NJ, Locascio JJ, Rentz DM, Johnson KA (2012). Neuropsychiatric symptoms and global functional impairment along the Alzheimer's continuum. Dement Geriatr Cogn Disord.

[21] Koivisto K, Reinikainen KJ, Hanninen T, Vanhanen M, Helkala EL, Mykkanen L (1995). Prevalence of age-associated memory impairment in a randomly selected population from eastern Finland. Neurology.

[22] Petrides M (1991). Monitoring of selections of visual stimuli and the primate frontal cortex. Proc Biol Sci.

[23] Callicott JH, Bertolino A, Mattay VS, Langheim FJ, Duyn J, Coppola R (2000). Physiological dysfunction of the dorsolateral prefrontal cortex in schizophrenia revisited. Cereb Cortex.

[24] Kaasinen V, Nurmi E, Bruck A, Eskola O, Bergman J, Solin O (2001). Increased frontal [(18)F]fluorodopa uptake in early Parkinson's disease: sex differences in the prefrontal cortex. Brain.

[25] Durston S, Mulder M, Casey BJ, Ziermans T, van Engeland H (2006). Activation in ventral prefrontal cortex is sensitive to genetic vulnerability for attention-deficit hyperactivity disorder. Biol Psychiatry.

[26] Gong YS, Guo J, Hu K, Gao YQ, Xie BJ, Sun ZD (2016). Ameliorative effect of lotus seedpod proanthocyanidins on cognitive impairment and brain aging induced by D-galactose. Exp Gerontol.

[27] Chang L, Liu X, Liu J, Li H, Yang Y, Liu J (2014). D-galactose induces a mitochondrial complex I deficiency in mouse skeletal muscle: potential benefits of nutrient combination in ameliorating muscle impairment. J Med Food.

[28] Zhang XL, An LJ, Bao YM, Wang JY, Jiang B (2008). d-galactose administration induces memory loss and energy metabolism disturbance in mice: protective effects of catalpol. Food Chem Toxicol.

[29] Lye JJ, Latorre E, Lee BP, Bandinelli S, Holley JE, Gutowski NJ (2019). Astrocyte senescence may drive alterations in GFAPalpha, CDKN2A p14(ARF), and TAU3 transcript expression and contribute to cognitive decline. Geroscience.

[30] Csipo T, Lipecz A, Ashpole NM, Balasubramanian P, Tarantini S (2020). Astrocyte senescence contributes to cognitive decline. Geroscience.

[31] Tarantini S, Yabluchanskiy A, Csipo T, Fulop G, Kiss T, Balasubramanian P (2019). Treatment with the poly(ADP-ribose) polymerase inhibitor PJ-34 improves cerebromicrovascular endothelial function, neurovascular coupling responses and cognitive performance in aged mice, supporting the NAD+ depletion hypothesis of neurovascular aging. Geroscience.

[32] Csiszar A, Yabluchanskiy A, Ungvari A, Ungvari Z, Tarantini S (2019). Overexpression of catalase targeted to mitochondria improves neurovascular coupling responses in aged mice. Geroscience.

[33] Wei H, Li L, Song Q, Ai H, Chu J, Li W (2005). Behavioural study of the D-galactose induced aging model in C57BL/6J mice. Behav Brain Res.

[34] Zhu J, Mu X, Zeng J, Xu C, Liu J, Zhang M (2014). Ginsenoside Rg1 prevents cognitive impairment and hippocampus senescence in a rat model of D-galactose-induced aging. PLoS One.

[35] Zhu G, Wang Y, Li J, Wang J (2015). Chronic treatment with ginsenoside Rg1 promotes memory and hippocampal long-term potentiation in middle-aged mice. Neuroscience.

[36] Chen L, Yao H, Chen X, Wang Z, Xiang Y, Xia J (2018). Ginsenoside Rg1 Decreases Oxidative Stress and Down-Regulates Akt/mTOR Signalling to Attenuate Cognitive Impairment in Mice and Senescence of Neural Stem Cells Induced by D-Galactose. Neurochem Res.

[37] Johnson SC, Rabinovitch PS, Kaeberlein M (2013). mTOR is a key modulator of ageing and age-related disease. Nature.

[38] Nie L, Xia J, Li H, Zhang Z, Yang Y, Huang X (2017). Ginsenoside Rg1 Ameliorates Behavioral Abnormalities and Modulates the Hippocampal Proteomic Change in Triple Transgenic Mice of Alzheimer's Disease. Oxid Med Cell Longev.

[39] Chen WF, Zhou LP, Chen L, Wu L, Gao QG, Wong MS (2013). Involvement of IGF-I receptor and estrogen receptor pathways in the protective effects of ginsenoside Rg1 against Abeta(2)(5)(-)(3)(5)-induced toxicity in PC12 cells. Neurochem Int.

[40] Chan RY, Chen WF, Dong A, Guo D, Wong MS (2002). Estrogen-like activity of ginsenoside Rg1 derived from Panax notoginseng. J Clin Endocrinol Metab.

[41] Alzheimer's A (2013). 2013 Alzheimer's disease facts and figures. Alzheimers Dement.

[42] Mossello E, Ballini E (2012). Management of patients with Alzheimer's disease: pharmacological treatment and quality of life. Ther Adv Chronic Dis.

[43] Gandy S, DeKosky ST (2013). Toward the treatment and prevention of Alzheimer's disease: rational strategies and recent progress. Annu Rev Med.

[44] Lee CH, Kim JH (2014). A review on the medicinal potentials of ginseng and ginsenosides on cardiovascular diseases. J Ginseng Res.

[45] Gao Y, Chu S, Zhang Z, Chen N (2017). Hepataprotective effects of ginsenoside Rg1 - A review. J Ethnopharmacol.

